# Unraveling Adipocytes and Cancer Links: Is There a Role for Senescence?

**DOI:** 10.3389/fcell.2020.00282

**Published:** 2020-04-28

**Authors:** Qi Wu, Bei Li, Si Sun, Shengrong Sun

**Affiliations:** ^1^Department of Breast and Thyroid Surgery, Renmin Hospital of Wuhan University, Wuhan, China; ^2^Faculty of Medicine, University of Paris Sud-Saclay, Le Kremlin-Bicêtre, France; ^3^Department of Clinical Laboratory, Renmin Hospital of Wuhan University, Wuhan, China

**Keywords:** adipocytes, cancer, senescence, tumor microenvironment (TEM), inflammation

## Abstract

Senescence is characterized by a permanent cell cycle arrest that is elicited in response to different stresses. In addition, senescent cells undergo multiple other phenotypic alterations, such as autophagy modulation, metabolic reprogramming, and the senescence-associated secretory phenotype (SASP). These senescence-related and inflammatory effects prevail within tumors and are strongly controlled by cancer properties, and inflammatory mediators further maintain and propagate the senescence process to adjacent cells. It is important to consider these detrimental effects that may drive tumorigenesis or cancer relapse. Importantly, cancer-associated adipocytes (CAAs) are one of the primary stromal cells in various tumor microenvironments and favor tumor progression by releasing various factors that can mediate local and systemic effects. However, it remains unclear whether CAAs possess senescent features. In this review, we discuss the complex relationship between senescence and CAAs and highlight important considerations for therapeutics.

## Introduction

Adipocytes are one of the primary stromal cells present in multiple cancers, such as breast, ovarian and colorectal cancer (Nieman et al., 2011; Wu et al., 2019a). Specifically, cancer-associated adipocytes (CAAs) are considered to play an active role in the tumor microenvironment. CAAs are capable of exhibiting several phenotypes, which, in aggregate, define their state. Morphologically, CAAs have a decreased cell size and a dilated interstitial space and exhibit a dramatic loss of lipid content (Lapeire et al., 2014). In terms of specific markers, CAAs show a reduction in adipose markers such as hormone-sensitive lipase, resistin, and adipocyte fatty acid-binding protein but a remarkable increase in fibroblast-like biomarkers such as fibroblast activation protein α, chondroitin sulfate proteoglycan, and α smooth muscle actin (Jung et al., 2015). Compared to other cells CAAs secrete more chemokine (C–C motif) ligand 2 (CCL2), chemokine (C–C motif) ligand 5 (CCL5), interleukin-6 (IL-6), tumor necrosis factor-alpha, vascular endothelial growth factor (VEGF), and leptin but less adiponectin (Dirat et al., 2011; Wu et al., 2019a). In terms of metabolic changes, CAAs enhance various catabolic processes and release high-energy metabolites, such as lactate, pyruvate, free fatty acids, and ketone bodies (Wu et al., 2019a). Interestingly, skeletal muscle cells are induced to undergo apoptosis via tumor-derived microvesicles (He et al., 2014), while the adipocytes surrounding the tumor cells present smaller sizes with a dilated interstitial space (Dirat et al., 2011). Hence, CAAs possess substantial remodeling capacity but can still survive while under attack from tumor cells. The potential conversion of normal adipocytes to CAAs deserves further exploration.

Cellular senescence was originally identified as stable growth arrest, and it is further thought to be a stress response that can be triggered by a variety of intrinsic and extrinsic types of damage, such as oncogenic activation, oxidative and genotoxic stress, mitochondrial dysfunction, irradiation, and chemotherapeutic agents (Herranz and Gil, 2018). Many phenotypic alterations that are relevant to senescent processes are associated with the pathophysiological effects of senescent cells. For instance, senescent cells undergo a series of alterations, such as morphological changes, chromatin remodeling, and metabolic reprogramming, culminating in the senescence-associated secretory phenotype (SASP), which is described as the complex secretion of mostly proinflammatory factors (Rodier and Campisi, 2011). In addition, senescent cells in the surrounding microenvironment have tumor-promoting effects. In coculture experiments, senescent fibroblasts have been shown to regulate the growth of prostate epithelial cells with mutations, inducing the generation of preneoplastic cells (Bavik et al., 2006). Interestingly, senescent cells within the microenvironment and their functionally analogous cousins, cancer-associated fibroblasts (CAFs), promote transformation by stimulating tumor growth, angiogenesis, and invasion (Ruhland et al., 2016; Milanovic et al., 2018). The broad actions of CAFs and senescent cells in tumor progression have been ascribed to the plethora of tumorigenic factors that these cell types secrete (Alspach et al., 2013). However, whether CAAs in the tumor microenvironment undergo senescent transformation or possess some senescent characteristics remains unclear.

Adipose tissue plays a central role in longevity and age-related disorders (Tchkonia et al., 2010). The dysfunction of senescent fat tissues contributes to age-related chronic inflammation, diabetes, and cancer (Martyniak and Masternak, 2017; Sieben et al., 2018; Ghosh et al., 2019). Recent evidence has indicated that fat cells can be switched into a proinflammatory, dysfunctional, senescent-like state in individuals with obesity (Tchkonia et al., 2010; Ghosh et al., 2019). However, this would result in a significant reduction in the amount of proinflammatory cytokines (CCL2 and IL-6) derived from the visceral adipose tissue (VAT) of aging mice and decreases in the levels of cyclin-dependent kinases (p16 and p21) cultured in fetal-derived serum (Ghosh et al., 2019). This suggests that fetal plasma rescues the senescent phenotypes of VAT in old mice. Additionally, cellular senescence is regarded as an adaptive response to stress during which multiple cellular functions, including metabolic processes and secretome activity, have been readjusted (Herranz and Gil, 2018). Furthermore, autophagy and endoplasmic reticulum (ER) stress are two of the major cellular stress responses (Galluzzi et al., 2018). It is evident that increased expression of ER stress markers such as GRP78 and CHOP is observed in the adipose tissue of old mice, and activation of the ER stress response contributes to the elevated secretion in CCL2 and IL-6 (Ghosh et al., 2015). Likewise, autophagic function is diminished in the adipose tissue of aging mice, and impaired autophagy further facilitates ER stress (Ghosh et al., 2016). Taken together, these data indicate that disorganized stress responses destroy adipose tissue homeostasis and exacerbate age-associated inflammation. Here, we speculate the feasible molecular mechanisms that connect CAAs with cellular senescence.

## Autophagy

Autophagy is a mechanism for isolating and degrading various cytoplasmic structures, such as damaged organelles and invasive microorganisms, via lysosomes. Activated autophagy in the stroma promotes cancer progression. For example, high expression of Beclin-1, an inducer of autophagy, promotes the growth of fibroblasts in breast cancer (Capparelli et al., 2012). For human fibroblasts, activated autophagy results in their senescence (Young et al., 2009); meanwhile, CAFs from multiple cancers are found to exhibit senescent phenotypes (Dou et al., 2017). This evidence demonstrates that autophagy may play a crucial role in the malignant transformation and senescence of stromal cells. Moreover, there are several autophagy-associated signals in the stroma connecting senescence and the malignant transformation of adipocytes. First, caveolin-1 (Cav-1), a main structural protein of caveolae, plays an important role in membrane transport (endocytosis and transcytosis), the maintenance of membrane lipid composition and signal transduction within cells (Wang et al., 2017). Intriguingly, Cav-1 is widely considered a tumor suppressor, and low expression of Cav-1 in surrounding adipocytes drives tumor growth and metastasis in breast cancer (Witkiewicz et al., 2009; Wu et al., 2019b). Under hypoxic conditions, Cav-1 can be degraded in the autophagic response via activation of hypoxia inducible factor-1α (HIF-1α) and NF-κB (Martinez-Outschoorn et al., 2010). Recently, Cav-1 downregulation has been shown to contribute to the induction of cellular senescence in fibroblasts; Cav-1 deficiency impaired mitochondrial respiration and inactivated silent information regulator 2 homolog 1 (SIRT1) to promote premature senescence (Yu et al., 2017). Therefore, Cav-1-induced autophagy may be the crucial link between CAAs and cellular senescence. In addition, transforming growth factor β (TGF-β) is a multifunctional cytokine that not only modulates the growth and differentiation of cancer cells but also mediates the senescence of cancer stroma and autophagy (Kiyono et al., 2009; Hassona et al., 2013). Furthermore, TGF-β activates autophagy by inducing the accumulation of autophagosomes and converting microtubule-associated protein 1 light chain 3 to its lipid-modified form to promote the degradation of proteins (Kiyono et al., 2009). In addition, TGF-β accelerates fibroblast senescence by increasing reactive oxygen species (ROS) levels to reinforce malignant behavior (Hassona et al., 2013). TGF-β overexpression in conditioned media from breast cancer cells promoted a reversal of adipose cells to a fibroblastic phenotype (Guerrero et al., 2010). These results reveal the functional diversity of CAAs, suggesting that CAAs may enhance malignant behavior by inducing the senescence of adipocytes via TGF-β-dependent autophagy. Ultimately, the link between CAAs and senescence has been demonstrated in adipocytes with overexpression of HIF-1α, which facilitates autophagy and tumor growth (Diedrich et al., 2016; Wu et al., 2019b). Likewise, von Hippel-Lindau (VHL), a tumor suppressor gene, is a major regulator of HIF-1α subunits under aerobic conditions, and it directs proteasome-mediated degradation to inactivate HIF-1α function. It has been found that the absence of VHL induces senescence via regulation of p400 and p27, both of which enhance the ability of pRB to promote cell cycle arrest and senescence; this activity occurs in a HIF-α-dependent manner (Young et al., 2008).

**FIGURE 1:**
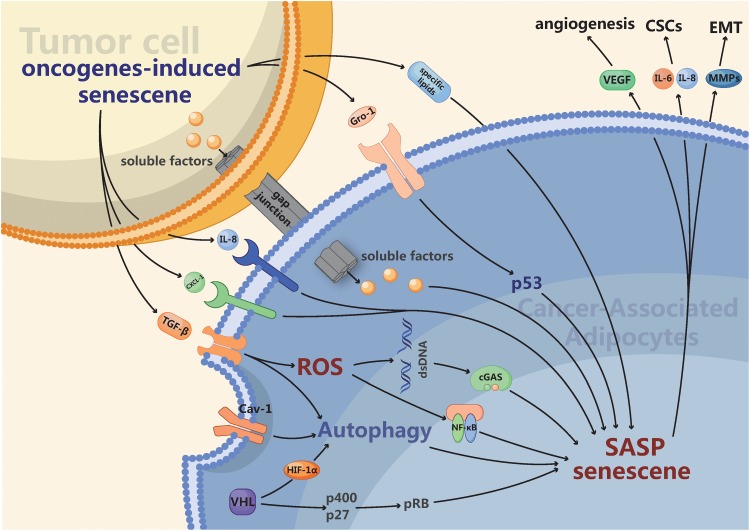
The intrinsic and extrinsic mechanisms connecting cancer-associated adipocytes (CAAs) with senescence. Firstly, Autophagy activated by diverse intracellular and extracellular stress like hypoxia in CAAs is a hallmark of senescence as well as a tumor promoter. Furthermore, the secreted profile of CAAs is enriched in many of the same pro-inflammatory factors, such as IL-6, IL-8, and a variety of chemokines, which overlap strongly with the senescence-associated secretory phenotype (SASP). Finally, the activation of several oncogenes such as RAS or the loss of tumor suppressors like p53 can promote senescence. The senescent CAAs may arise from gap junctions and paracrine signals originating from tumor cells or other senescent cells. Ultimately, CAAs possessing multifarious senescent properties may enhance tumor angiogenesis, proliferation of cancer stem cells (CSCs) and epithelial-mesenchymal transition (EMT).

In summary, studying autophagy in CAAs enables us to recognize the link between autophagy and senescence among these different compartments (Figure 1). In certain cancer subtypes, the activation of autophagic flux in the stroma is an indicator of senescence as well as a tumor promoter. Drugs that target autophagy or senescence are effective antitumor agents, and determining the autophagy or senescence status in the stroma may function as a potential biomarker.

## SASP

The SASP suggests that senescent cells communicate with neighboring cells to spread the stress response and influence their microenvironment. The SASP increases the expression of a range of chemokines (chemoattractants and macrophage inflammatory proteins), proinflammatory cytokines (most notably IL-1β, IL-6, and IL-8), growth factors (TGF-β, granulocyte-macrophage colony-stimulating factor, and hepatocyte growth factor), and matrix-remodeling enzymes (Lasry and Ben-Neriah, 2015; Herranz and Gil, 2018). Interestingly, recent work has shown that prior to the arrival of metastatic cells at a distal site, numerous changes that prepare the site for the growth of the tumor cells occur. Outstanding questions in this process include how the microenvironment changes at these distal sites and what cell types are responsible for these changes. Because senescent cells accumulate in tissue with age (Krtolica et al., 2001), these observations raise the possibility that senescent cells within the microenvironment may have the potential to condition the pre-metastatic niche and increase the likelihood of the establishment of metastases. Moreover, attention has been paid to investigating the underlying mechanisms that induce the appearance of cancer-initiating cells or cancer stem cells (CSCs), which seem to drive the formation of distant metastasis (Batlle and Clevers, 2017). Intriguingly, chemokines secreted by senescent cells have been shown to select for CSCs (Milanovic et al., 2018). As one of the most strongly expressed SASP factors, IL-6 has crucial effects in regulating the self-renewal of breast CSCs (Korkaya et al., 2011). Similarly, analyses of breast CAFs recently revealed CCL2 expression can stimulate CSC-like properties, including increased sphere-forming capacity and self-renewal activity (Tsuyada et al., 2012; Milanovic et al., 2018). The interleukins IL-6 and IL-8 facilitate the growth of breast cancer epithelial cells. Treatment with neutralizing antibodies against IL-6 and IL-8 decreased the promotion of growth of preneoplastic epithelial cells cocultured with senescent cells (Coppe et al., 2008). Furthermore, treatment with recombinant IL-6 and IL-8 could effectively promote the growth of breast cancer epithelial cells (Ortiz-Montero et al., 2017). Moreover, senescent cells could facilitate epithelial-to-mesenchymal transition (EMT), which plays an important role in tumor cell metastasis. Previous work has demonstrated that after treatment with conditioned media from senescent fibroblasts, human breast cancer cell lines showed decreased expression of cytokeratin and E-cadherin, which are hallmarks of EMT (Coppe et al., 2008). Senescent cells induce the upregulation of EMT via matrix metalloproteinase 3 (Parrinello et al., 2005). In addition, senescent fibroblasts promote the invasion of human umbilical vascular endothelial cells (HUVECs) *in vitro* and facilitate the vascularization of tumors in xenograft experiments by secreting VEGF (Coppe et al., 2006). Ultimately, sFRP2, as a Wnt antagonist, can be secreted by aging fibroblasts. sFRP2 promotes the loss of APE1, a key redox effector, in melanoma cells by decreasing the levels of β-catenin and microphthalmia-associated transcription factor (MITF). Furthermore, the increase in sFRP2 endows melanoma cells with the capacity to resist targeted therapy and metastasis (Kaur et al., 2016). In conclusion, these results reveal that senescent cells promote the establishment of primary tumors by expressing SASP factors.

Similar to senescent fibroblasts within the tumor microenvironment, CAAs stimulate tumor growth, angiogenesis, invasion, and metastasis to facilitate the transformation process (Dirat et al., 2011; Lapeire et al., 2014; Wu et al., 2019a). The tumor-promoting activity of CAAs is partially mediated by an altered secretion phenotype that overlaps strongly with the SASP (Figure 1). Indeed, several studies have demonstrated that the expression profile of CAAs is enriched in many of the same proinflammatory factors, such as IL-6, IL-8, and a variety of CCLs, that are present in the SASP (Dirat et al., 2011; Vazquez Rodriguez et al., 2018; Wu et al., 2019b). Thus, it was not surprising that similar to senescent cells, CAAs also express increased levels of MMPs (Dirat et al., 2011; Rowan et al., 2014), which can enhance EMT to promote tumor metastasis. In addition, adipose-derived stem cells treated with tumor-derived extracellular vesicles could secrete more VEGF to promote angiogenic sprouting of HUVECs (Song et al., 2017). Given the phenotypic similarities and emerging molecular similarities between senescent cells and CAAs, we have argued that CAAs may be an operational subtype of senescent cells.

## Oncogene-Induced Senescence

Tumor-transformed adipocytes have been demonstrated to form via the expression of various tumor-derived inflammatory factors and contain multiple exosomes (Petruzzelli et al., 2014; Wu et al., 2018, 2019c). Likewise, in response to the activation of many oncogenes, normal cells must undergo cellular senescence. For example, when an oncogenic form of RAS was expressed in human fibroblasts, oncogene-induced senescence (OIS) was originally observed (Serrano et al., 1997). The number of oncogenes able to induce senescence has risen to ∼50 oncogenes (Gorgoulis and Halazonetis, 2010). In addition, the loss of tumor suppressors can promote senescence, such as the loss of PTEN (Alimonti et al., 2010), P53 (Acosta et al., 2013), or VHL (Young et al., 2008). Cellular senescence can also be induced by established tumors or neighboring neoplastic cells, as demonstrated by a study in which senescence was induced in the stroma surrounding tumors through injection of tumor cells (Burd et al., 2013). Mechanistically, OIS can induce senescence of neighboring cells through induction of SASP or gap junction-mediated cell-cell contact, thereby enhancing its own effects (Nelson et al., 2012; Acosta et al., 2013). Indeed, several studies have demonstrated that numerous SASP factors, including TGF-β(Acosta et al., 2013), IL-8, and CXCL1 (Acosta et al., 2008), and several SASP pathways, such as NF-κB signaling activated by ROS (Nelson et al., 2018) and cGAS–STING signaling (Yang et al., 2017), can mediate the induction of paracrine senescence. Cancer cells produce ROS and increase oxidative stress in adjacent adipocytes (Ladanyi et al., 2018), and ROS are necessary and sufficient to activate NF-κB, which promotes SASP and results in the DNA damage response in CAAs (Nelson et al., 2018). Moreover, with the accumulation of DNA products from impaired synthesis in the cytoplasm, cGAS, as a cytosolic DNA sensor, is essential for senescence (Yang et al., 2017). In addition, chemokine growth-regulated oncogene 1 (Gro-1) was activated by RAS and upregulated in serum samples from ovarian cancer patients. Furthermore, Gro-1 could facilitate the senescence of stromal fibroblasts by affecting functional p53 to promote tumor growth (Yang et al., 2006). Extracellular vesicles also play an important role in paracrine signaling by transferring contents to impact recipient cell signaling. A recent study indicated that extracellular vesicles derived from cells with H-Ras-induced senescence were enriched and capable of transporting multiple specific lipids, including hydroxylated sphingomyelin, sphingomyelin, lysophosphatidic acid, and sulfatides (Buratta et al., 2017). In summary, the paracrine senescence induced by OIS reveals a mechanism by which senescent adipocytes can gain enhanced effects and may amplify negative effects on tumors (Figure 1).

## Concluding Remarks and Future Directions

Cellular senescence, a typical characteristic of cancer, can be triggered by a variety of mechanisms in and around tumors and has deleterious effects on tumor initiation, growth, recurrence, and therapeutic efficacy. However, the senescent characteristics of adipocytes and the mechanisms by which they affect the phenotype of tumor cells remain incompletely understood. Indeed, autophagy, a central regulator in senescent cells, mediates the functions of CAAs. Therefore, further characterization of autophagy in CAAs and cancer is crucial for us to understand these mechanisms and develop targeted therapeutic approaches in the future. Additionally, it is important to again note the similarities between the SASP and the expression profile of CAAs. The overlap in expression profiles between these two tumor-promoting cell types suggests that the regulatory pathways that are further characterized in senescent cells will be directly applicable in CAAs. Given the potent tumor-promoting nature of the SASP, identifying the regulatory mechanisms that govern the expression of genes related to this phenotype will contribute to the development of stroma-targeting cancer therapies. This concept is consistent with our speculation that a subset of senescent CAAs is present and that OIS may be a major contributor to the generation of senescent adipocytes. The current challenge in the field is to discern which changes are critical for tumor progression and to determine how cell-autonomous mutations within incipient tumor cells influence stromal changes.

## AuthOr Contributions

QW and BL were responsible for collecting and collating documents. QW and BL were responsible for writing this review, while SiS was responsible for the revision and ShS was responsible for editing and submission. All authors read and approved the final manuscript.

## Conflict of Interest

The authors declare that the research was conducted in the absence of any commercial or financial relationships that could be construed as a potential conflict of interest.
